# Maintenance Chemotherapy in Patients with Platinum-Sensitive Relapsed Epithelial Ovarian Cancer after Second-Line Chemotherapy

**DOI:** 10.3390/jcm13020566

**Published:** 2024-01-18

**Authors:** Yen-Fu Chen, Shih-Tien Hsu, Sheau-Feng Hwang, Lou Sun, Chih-Ku Liu, Yu-Hsiang Shih, Ting-Fang Lu, Jun-Sing Wang, Chien-Hsing Lu

**Affiliations:** 1 Department of Obstetrics and Gynecology, Taichung Veterans General Hospital, Taichung 407219, Taiwan; tridone@vghtc.gov.tw (Y.-F.C.); sthsu@vghtc.gov.tw (S.-T.H.); sfhwng@vghtc.gov.tw (S.-F.H.); sunlou@vghtc.gov.tw (L.S.); mescal@vghtc.gov.tw (C.-K.L.); ssuuyy22@vghtc.gov.tw (Y.-H.S.); lu760509@vghtc.gov.tw (T.-F.L.); 2 Center for General Education, Ling Tung University, Taichung 408213, Taiwan; 3 School of Medicine, China Medical University, Taichung 404328, Taiwan; 4 Department of Palliative Care Unit, Taichung Veterans General Hospital, Taichung 407219, Taiwan; 5 Department of Medicine, School of Medicine, National Yang Ming Chiao Tung University, Taipei 112304, Taiwan; 6 Division of Endocrinology and Metabolism, Department of Internal Medicine, Taichung Veterans General Hospital, Taichung 407219, Taiwan; 7 College of Medicine, National Chung Hsing University, Taichung 402202, Taiwan; 8 Rong Hsing Research Center for Translational Medicine, National Chung Hsing University, Taichung 402202, Taiwan

**Keywords:** recurrent ovarian cancer, platinum-sensitive ovarian cancer, epithelial ovarian cancer, chemotherapy, maintenance therapy

## Abstract

(1) Background: Our aim was to evaluate the efficacy and adverse effects of maintenance chemotherapy in platinum-sensitive recurrent epithelial ovarian cancer after second-line chemotherapy. (2) Methods: A total of 72 patients from a single institute who had been diagnosed with platinum-sensitive recurrent ovarian cancer and had experienced either complete or partial response after six cycles of second-line chemotherapy were divided into a standard group (*n* = 31) with six cycles or a maintenance group (*n* = 41) with more than six cycles. We then compared patient characteristics and survival outcomes between these two groups. (3) Results: In all patients, after primary management for the first recurrence, the maintenance group showed worse survival outcomes. Patients who had not undergone either surgery or radiotherapy were divided into complete response and partial response groups after six cycles of chemotherapy. In patients with partial response, maintenance chemotherapy led to a significant improvement in PFS (median, 3.6 vs. 6.7 months, *p* = 0.007), but no significant change in in OS. The median cycle number of maintenance chemotherapy was four. (4) Conclusions: Maintenance chemotherapy may still play an important role in patients with platinum-sensitive recurrent ovarian cancer, particularly in selected patient groups.

## 1. Introduction

Epithelial ovarian cancer (EOC) has been one of the leading causes of death in women worldwide over the past decade, with over 314,000 women being diagnosed in the year 2020 alone, and approximately 207,000 of them dying, making it the second most common cause of death from gynecologic cancers [1]. In Taiwan, an incidence rate of 6.36 per 100,000 women in 2020 was documented, with a steady increase since the 1990s at an annual percentage change of 1.5% during the period 1995 to 2014 [2]. Ovarian cancer is also one of the most common causes of death in women, with a standardized mortality rate of 3.3 per 100,000 women being seen in the year 2021. Approximately 70% of EOC patients present with advanced-stage disease, with the standard treatment consisting of cytoreductive surgery followed by platinum-based chemotherapy [3]. Although platinum-based therapy is initially effective in most patients, the majority will experience disease recurrence within the first two years, and only a minority will achieve long-term remission. Furthermore, its platinum-free interval becomes shorter with every incidence of recurrence.

Recently, the use of poly-adenosine diphosphate–ribose polymerase (PARP) inhibitors and bevacizumab has become mainstream first-line maintenance management treatment of ovarian cancer, including even in a recurrent setting [4,5,6,7]. While many countries have incorporated the treatment into their routine method of care, there are still certain countries that do not offer sufficient insurance reimbursements for these agents, leaving many patients unable to personally afford the treatment due to financial limitations. Therefore, chemotherapy remains the key element in the recurrent setting in many countries.

The number of cycles required during first-line adjuvant chemotherapy has been previously evaluated [8,9,10], however, the role of maintenance chemotherapy in first-line management remains controversial [10,11,12]. Based on the results from several trials [13,14,15], six cycles of first-line adjuvant chemotherapy were found to be adequate, with extended cycles not showing any survival benefits, but rather more adverse effects. However, in the platinum-sensitive recurrent setting, defined as when patients have a platinum-free interval (PFI) of more than 6 months after the completion of their last platinum-based regimen, there is still a lack of robust evidence on the number of cycles necessary during second-line platinum-based chemotherapy [16,17,18]. Previous studies focused mainly on the chemotherapy agents instead of the relationship between the number of cycles and survival benefits, and most of them kept six to nine cycles without addressing the role of extended cycles. 

According to the National Comprehensive Cancer Network (NCCN) guidelines, six cycles of platinum-based doublet chemotherapy is recommended, no matter whether the status of the disease differs between patients. Therefore, whether a complete or partial response is reached after six cycles poses the question: Do better survival outcomes result from maintenance chemotherapy?

In this retrospective study, we sought to validate the efficacy of maintenance chemotherapy in its role of prolonging progression-free survival, while also evaluating its adverse effects in patients with platinum-sensitive recurrent ovarian cancer in a tertiary medical center in Taiwan.

## 2. Materials and Methods

### 2.1. Study Population and Data Collection

This is a retrospective cohort study with data taken from a tertiary medical center in Taichung City, Taiwan. The study protocol was approved by our Institutional Review Board (IRB) (Number: CE21233B).

**Figure 1:**
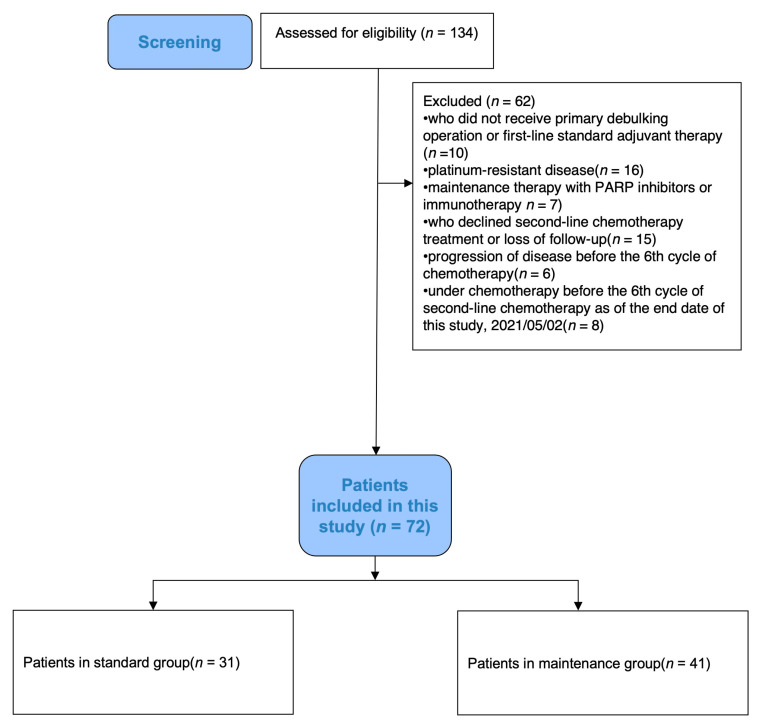
Patient selection flow chart.

We identified patients who had experienced their first relapse of epithelial ovarian cancer (EOC) in a decade between the years 2011 and 2021 (*n* = 134, Figure 1), with or without having undergone secondary cytoreduction surgery or radiation therapy. We excluded patients who had not received either a primary debulking operation or first-line standard adjuvant therapy at our medical center (*n* = 10), as well as patients with platinum-resistant disease (*n* = 16) as a recurrent disease less than 6 months from their last first-line chemotherapy session. Patients who opted for maintenance therapy involving PARP inhibitors or immunotherapy (*n* = 7) were excluded, however, those treated with bevacizumab were included. In addition, patients who declined standard chemotherapy treatment or were lost during follow-up (*n* = 15), patients who experienced disease progression before their 6th cycle of second-line chemotherapy (*n* = 6), and patients who were currently under chemotherapy before the evaluation of their 6th cycle of second-line chemotherapy as of the end date of this study on 2 May 2021 (*n* = 8) were also excluded. 

A total of 72 patients who had exhibited either complete or partial response after six cycles of second-line chemotherapy, based on the images from their CT scan and the incorporated criteria of RECIST 1.1 and CA-125 agreed by the Gynecological Cancer Intergroup (GCIG), were included in the study. The patients were then divided into two groups according to the number of cycles they received, which included both a standard group (6 cycles) and a maintenance group (more than 6 cycles). Patients who underwent secondary cytoreduction surgery were evaluated first in multidisciplinary team meetings and needed to fulfill the following criteria: single or countable tumor lesions that were resectable, minimal ascites, and no distant metastases seen on PET or CT scans.

The status of disease progression and survival was confirmed by the date of 2 May 2021. Either an increase in the volume of tumors, the presence of new lesions or more ascites on abdominal CT scans, or an elevation in CA-125 over twice the upper limit of the response range was defined as disease progression, which was 70 IU/mL at our institution. The primary outcome of this study was progression-free survival (PFS) and overall survival (OS) as a secondary endpoint in terms of maintenance therapy. To explore the role of maintenance chemotherapy, PFS was defined as the interval from the date of the sixth cycle of second-line chemotherapy to the date of disease progression or patient mortality. OS was defined as the interval from the date of the sixth cycle of second-line chemotherapy to the date of mortality.

### 2.2. Statistical Analysis

Differences in baseline characteristics between the two treatment groups were compared using the Mann–Whitney U test and chi-square test for continuous and categorical variables, respectively. The Kaplan–Meier method was used to generate survival curves for OS and PFS, while Cox proportional hazard analysis was performed to determine the association of maintenance chemotherapy treatment with OS and PFS after adjustments for relevant variables. Subgroup analysis was conducted to determine whether a significant variation existed in the aforementioned association. All statistical analyses were performed using the Statistical Package for Social Sciences (IBM SPSS version 22.0; International Business Machines Corp, Armonk, NY, USA) with a *p* value of <0.05 considered as being statistically significant.

## 3. Results

Overall, 72 patients were included in this analysis: 41 in the maintenance group (>6 cycles) and 31 in the standard group (6 cycles). To better evaluate the effect of maintenance chemotherapy, we compared patients in groups with secondary cytoreduction surgery or chemotherapy alone. We found that only in the subgroup of patients who did not receive secondary cytoreduction surgery and had partial response after six cycles did they present a significant improvement in progression-free survival (PFS). However, we carried out a thorough analysis of every group in the attempt to explore which subgroup of patients would benefit most from the maintenance chemotherapy. 

### 3.1. Analysis of All Patients (n = 72)

Table 1 presents the clinicopathologic characteristics of all patients. No differences in patient age, histologic type, tumor grade, FIGO stage, or primary treatment strategy (chemotherapy regimens) were observed between the maintenance and standard groups. Most of the patients had been diagnosed with serous ovarian cancer (58/72, 80.5%) at the advanced stage (59/72, 81.9% stage III–IV) during initial diagnosis. Nearly all of the patients underwent optimal debulking surgery (64/72, 88.8%), with 17 of them (17/72, 23.6%) receiving neoadjuvant chemotherapy. All of the patients were treated with a combination of paclitaxel and carboplatin as first-line adjuvant chemotherapy. The platinum-free interval (PFI) on average was significantly longer in the standard group than that seen in the maintenance group (median, 19.4 vs. 13.9 months, *p* = 0.038). Additionally, there was a significantly higher level of serum CA-125 in the maintenance group at first recurrence (median, 181.5 vs. 68.1 IU/mL; *p* = 0.005). In all patients (*n* = 72), median progression-free survival (PFS) was 15.3 months, while overall survival (OS) was 52.1 months. 

**Figure 2:**
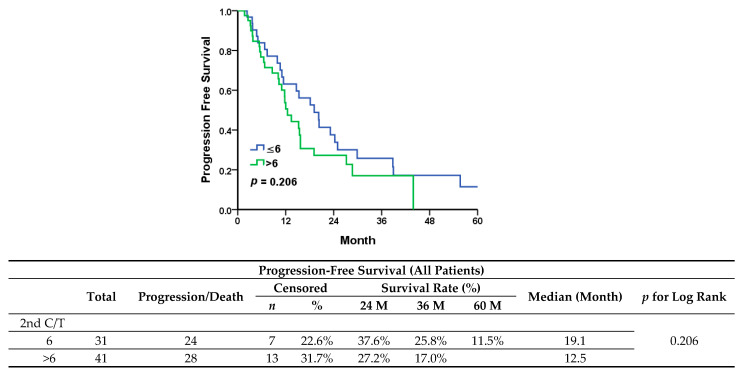
Progression-free survival in all patients.

**Figure 3:**
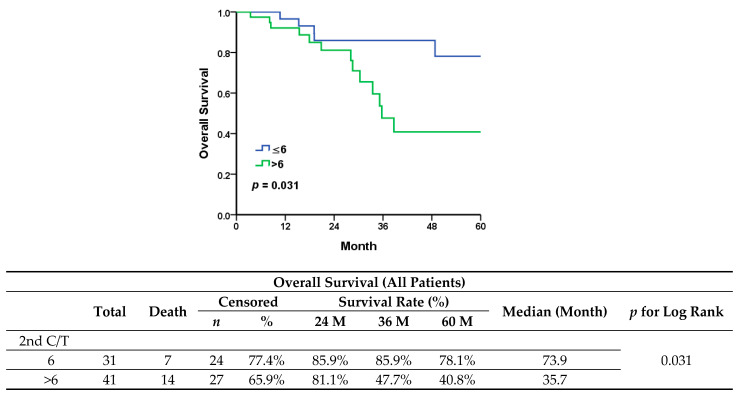
Overall survival in all patients.

After the first recurrence, 26 (36.1%) patients underwent secondary cytoreductive surgery, with complete gross resection (R0) achieved in all of them. Two patients who had received radiotherapy were identified with localized recurrence but could not be resected completely, one of whom was diagnosed with brain metastases and the other with paraaortic lymphadenopathy, before the initiation of second-line chemotherapy. There were 44 patients (44/72, 61.1%) who underwent second-line salvage chemotherapy only. After primary management for first recurrence, the maintenance group showed worse outcomes in progression-free survival (Figure 2) (median, 12.5 vs. 19.1 months; *p* = 0.206) and significantly shorter overall survival (Figure 3) (median, 35.7 vs. 73.9 months; *p* = 0.031).

In the standard group (*n* = 31), all of the patients received six cycles of chemotherapy, with the most common regimen being platinum plus pegylated liposomal doxorubicin (PLD) (*n* = 16, 52%), followed by platinum plus paclitaxel (*n* = 14, 45%). The number of patients who received bevacizumab as maintenance therapy after second-line chemotherapy showed no significant difference between the two groups (51.2% vs. 38.7%, *p* = 0.291). In the maintenance chemotherapy group (*n* = 41), the median cycle number for second-line chemotherapy was nine (range 7–22). The most common regimen in the maintenance group was also platinum plus PLD (*n* = 25, 60.9%), followed by platinum plus paclitaxel (*n* = 11, 26.8%). Therefore, the chemotherapy regimens were mostly doublet platinum-based (87.8% vs. 96.8%, *p* = 0.227), which showed no difference between the two groups. More patients in the standard group received secondary cytoreduction surgery than those in the maintenance group (Table 1) (67.7% vs. 17%, *p* < 0.001), which correlates to a higher percentage being seen in patients with complete response after the 6th cycle of chemotherapy in the standard group (87.1% vs. 48.8%, *p* < 0.001). 

Multivariate analyses (Table 2), adjusting for variables such as complete response after six cycles of chemotherapy and PFI, showed that maintenance chemotherapy did not have an impact on either PFS (adjusted HR, 0.73; 95%CI, 0.34–1.58; *p* = 0.425) or OS (adjusted HR, 0.79; 95%CI, 0.22–2.81; *p* = 0.722). For OS, complete response after six cycles of chemotherapy was the only favorable prognostic factor with any significance (HR, 0.21; 95%CI, 0.05–0.92; *p* = 0.038) (Table 3). For PFS, a PFI ≥ 12 months was identified as the only significantly favorable prognostic factor (HR, 0.12; 95%CI, 0.05–0.29; *p* < 0.001) (Table 2).

### 3.2. Analysis of Patients Who Did Not Undergo Surgery or Radiotherapy (n = 44)

Table 4 demonstrates that no differences in patient age, histologic type, tumor grade, FIGO stage, or primary treatment strategy were observed between the maintenance and standard groups. Unlike the results seen in all patients, the PFI was of no significance between the two groups (median, 12.1 vs. 11.3 months, *p* = 0.481). There was, however, a significantly higher level of serum CA-125 at first recurrence in the maintenance group (median, 250.0 vs. 75.7 IU/mL; *p* = 0.033). During and after platinum doublet chemotherapy, the number of patients who received bevacizumab as maintenance therapy showed no significance (50.0% vs. 44.1%; *p* = 1.00).

**Figure 4:**
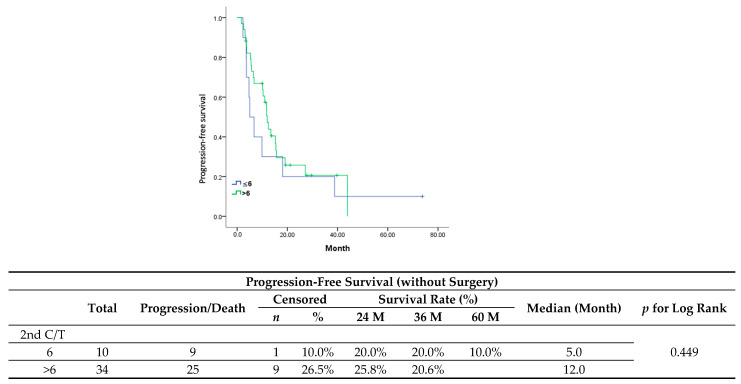
Progression-free survival in patients without surgery.

**Figure 5:**
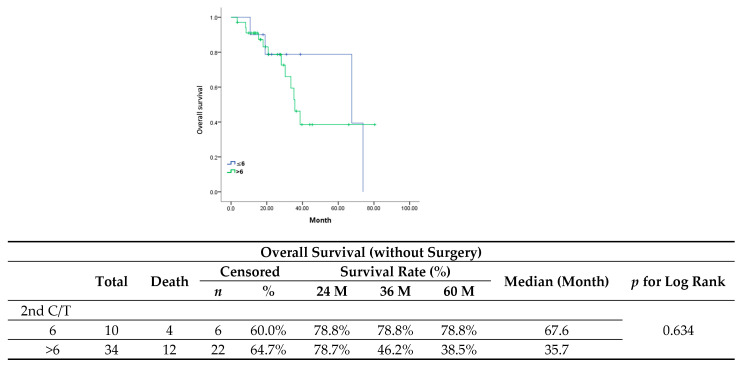
Overall survival in patients without surgery.

After primary management for the first recurrence, the maintenance group showed a poorer performance in PFS (Figure 4) (median, 5 vs. 12 months; *p* = 0.449) and OS (Figure 5) (median, 35.7 vs. 67.6 months; *p* = 0.634). Multivariate analyses, after adjusting for variables such as complete response after six cycles of chemotherapy and PFI, revealed that maintenance chemotherapy did not have an impact on either PFS (HR, 0.95; 95%CI, 0.43–2.07; *p* = 0.893) or OS (HR, 1.16; 95%CI, 0.37–3.62; *p* = 0.799) (Table 5 and Table 6). For PFS, a PFI ≥12 months was identified as the only favorable prognostic factor (HR, 0.18; 95%CI, 0.06–0.48; *p* = 0.001).

**Figure 6:**
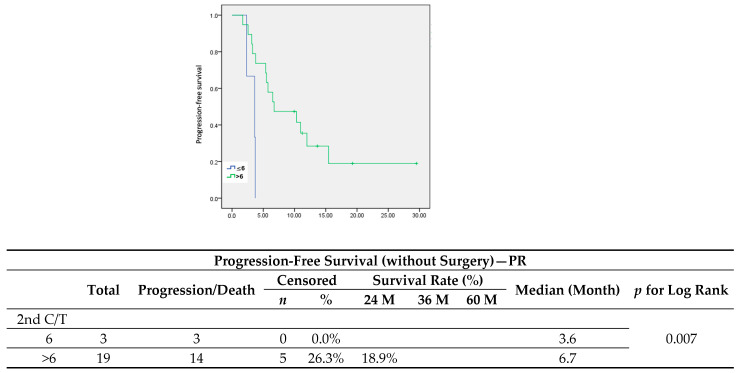
Progression-free survival in patients without surgery who had a partial response after 6 cycles of chemotherapy.

**Figure 7:**
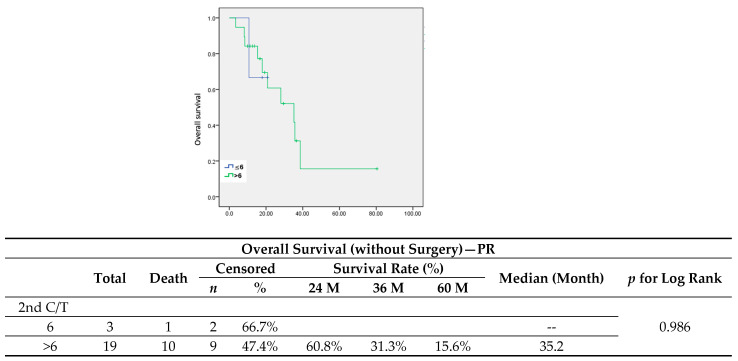
Overall survival in patients without surgery who had a partial response after 6 cycles of chemotherapy.

To better understand the role of maintenance chemotherapy, we focused on the patients who had not undergone surgery or radiotherapy and divided them more specifically into complete response and partial response groups after six cycles of second-line chemotherapy. In patients with partial response, maintenance chemotherapy led to a significant improvement in PFS (Figure 6) (median, 3.6 vs. 6.7 months, *p* = 0.007), but showed no significance in OS (Figure 7). The median cycle number of maintenance chemotherapy was 4 (range from 1–16).

### 3.3. Adverse Effects in All Patients

According to the criteria of CTCAE 5.0, adverse effects such as anemia, neutropenia, thrombocytopenia, liver toxicities, and renal toxicities were identified as both mild (grade 1–2) and severe (grade 3–4). Differences were only observed in mild anemia between the groups (51.6% vs. 75.6%, *p* = 0.034). The other adverse effects were of no significant difference between the two groups in terms of severity (Table 7).

## 4. Discussion

In our study, we evaluated maintenance chemotherapy in all patients diagnosed with platinum-sensitive recurrent ovarian cancer. In comparison to the standard chemotherapy group, the maintenance group presented a worse trend in progression-free survival (median, 12.5 vs. 19.1 months; *p* = 0.206) and significantly shorter overall survival (median, 35.7 vs. 73.9 months; *p* = 0.031). Since patients diagnosed with less extensive recurrent disease are more prone to receiving either secondary cytoreduction surgery or radiotherapy, a better outcome in these patients would be expected, therefore they would then receive only six cycles of adjuvant chemotherapy. All patients in the standard group showed a longer PFI and a significantly lower serum level of CA-125 at first recurrence. However, when it came to patients with a partial response after six cycles of second-line chemotherapy who had received only chemotherapy after recurrence (*n* = 21), maintenance chemotherapy led to a significant improvement in PFS (Figure 6) (median, 3.6 vs. 6.7 months, *p* = 0.007).

The essence of maintenance therapy lies in the prolonging of the platinum-free interval (PFI), which correlates to better survival outcomes and favorable use in platinum-based chemotherapy regimens. Maintenance chemotherapy has been studied with a single agent of paclitaxel, pegylated liposomal doxorubicin combined with carboplatin, or paclitaxel with carboplatin in first-line adjuvant therapy [8,10,12]. Here, mixed results were found, as some showed improvement in PFS, although no significant improvement was seen in OS among those studies. As for other maintenance agents, bevacizumab in first-line maintenance therapy only offered a benefit in PFS to patients with advanced-stage disease [19,20]. In patients with BRCA mutations or homologous recombination deficiency (HRD), PARP inhibitors provided a significant improvement in PFS [21,22].

Regarding the recurrent setting, many studies have focused on patients with platinum-sensitive disease, with pegylated liposomal doxorubicin showing promising results [23,24], though there remains a lack of larger randomized trials. As long as the toxicities are tolerable, we expect survival benefits, particularly in patients with partial or complete response. Bevacizumab works as a maintenance agent according to the OCEANS study and GOG-0213 study, both of which showed there was an improvement in PFS and OS for patients with platinum-sensitive recurrence [5,25]. PARP inhibitors can also be candidates for maintenance therapy in platinum-sensitive recurrence [6]. Maintenance chemotherapy still plays an important role, particularly in patients without BRCA mutations or homologous recombination deficiency (HRD), as well as patients in countries whose NHI does not cover the cost of the agents as mentioned above due to financial issues and the currently limited evidence among these populations [7]. 

Upon reviewing previous studies [24,26,27,28,29], controversies remain regarding the role of maintenance chemotherapy in patients with platinum-sensitive recurrent ovarian cancer. What we learned from previous studies validated the availability of platinum-based doublet chemotherapy regimens, which included platinum plus paclitaxel [18], platinum plus gemcitabine [30], and platinum plus pegylated liposomal doxorubicin [16]. The adequate cycle number in second-line chemotherapy has yet to be determined due to the lack of convincing results in the aforementioned studies, which suggested no survival benefits after six cycles, particularly in patients with complete response [8,11,31]. 

A recent retrospective study from Korea [31] analyzed the use of extended chemotherapy in patients who experienced platinum-sensitive recurrence after primary management with a residual tumor ≥0.5 cm (on CT scans) after six cycles of second-line, platinum-based chemotherapy. Under the same circumstance without secondary cytoreduction surgery, the characteristics of their patients were closely similar to our patients with partial response after six cycles of chemotherapy. In our study, there was significantly better PFS in the maintenance group (median, 6.7 vs. 3.6 months; *p* = 0.007). Compared to the data resulting from the Korean study, our results showed significantly worse PFS in the extended group (median, 13.9 vs. 14.8 months; *p* = 0.036). However, in the Korean study, patients without surgery who received extended cycles of chemotherapy were inferior to the standard group in several factors: fewer patients achieved R0 (41.2% vs. 56.7%) after primary debulking surgery, the platinum-free interval was shorter (median, 11.0 vs. 12.7 months), and fewer patients received any maintenance therapy (11.8% vs. 32.8%). Even under multivariate analysis, all the above factors may still play a part in the reasons behind worse outcomes in the extended group. Additionally, the evaluations of CT scans in the Korean study showed no clear evidence of any response after second-line chemotherapy, which also raised the concern of whether smaller lesions would be neglected in CT scans. In conclusion, the reasons that no compatible results were found in the Korean study may lie not only in the fundamental inferior patient characteristics seen in the extended group but also in the incomplete evaluation of chemotherapy response in patients, some of whom may be identified as either platinum-resistant or experiencing progression of disease after six cycles of second-line chemotherapy.

Nevertheless, several limitations do exist in our study. First and foremost, the sample size of our study population was small, particularly concerning those patients who had not undergone secondary cytoreduction surgery or radiation (*n* = 44), which was our main focus here. Therefore, there exists the possibility of selection bias and statistical issues. When we focused on patients with partial response (*n* = 22), there were only 3 in the standard group and 19 in the maintenance group. Secondly, even though we performed multivariate analyses for identifying possible confounders and subgroup analyses such as PFI and secondary cytoreduction surgery, the heterogeneity of the whole study population was never resolved, thus possibly requiring further prospective RCTs. Notwithstanding, we used standardized criteria for selecting our patients with complete response and partial response to chemotherapy to identify the role of maintenance chemotherapy, which is seldom described in previous studies on the same topic. The results we came up with have helped shed light on the promising direction that maintenance chemotherapy will take in platinum-sensitive recurrent ovarian cancer.

## 5. Conclusions

To sum up, even though we initiated our study with the attempt to prove maintenance chemotherapy could provide survival benefits in epithelial ovarian patients with platinum-sensitive recurrence, there was no significant improvement in the whole cohort. Nonetheless, in a subgroup of patients who did not undergo secondary cytoreduction surgery and experienced partial response after six cycles of chemotherapy, maintenance chemotherapy could significantly improve PFS. Maintenance target therapy and biological therapy have been thriving in the recurrent setting in recent years. However, maintenance chemotherapy still could play an important role due to the financial considerations in countries in which target and biological therapies are not reimbursed, and the concept of maintenance chemotherapy deserves further prospective trials to confirm our findings.

## Figures and Tables

**Table 1:** The clinicopathologic characteristics of all patients.

All Patients (*n* = 72)							
	2nd C/T = 6 (*n* = 31)		2nd C/T > 6 (*n* = 41)		All (*n* = 72)		*p* Value
Age at initial diagnosis, years	55.5	(48.3–64.6)	58.3	(49.8–62.4)	56.9	(49.3–62.5)	0.613
Age at first recurrence, years	58.2	(51.1–66.6)	60.6	(51.2–64.8)	60.1	(51.2–64.8)	0.781
Histologic type							0.537
Non-serous	5	(16.1%)	9	(22.0%)	14	(19.4%)	
Serous	26	(83.9%)	32	(78.0%)	58	(80.6%)	
FIGO stage							0.178
I–II	8	(25.8%)	5	(12.2%)	13	(18.1%)	
III	19	(61.3%)	25	(61.0%)	44	(61.1%)	
IV	4	(12.9%)	11	(26.8%)	15	(20.8%)	
Primary treatment							0.858
PDS	24	(77.4%)	31	(75.6%)	55	(76.4%)	
NAC	7	(22.6%)	10	(24.4%)	17	(23.6%)	
Results of initial DBK ^1^							0.674
Optimal	27	(87.1%)	37	(90.2%)	64	(88.9%)	
Suboptimal	4	(12.9%)	4	(9.8%)	8	(11.1%)	
Platinum-free interval, months	19.4	(13.5–34.2)	13.9	(7.6–25.7)	16.6	(8.9–28.6)	0.038 *
Median (range)							0.010 *
6–12	6	(19.4%)	20	(48.8%)	26	(36.1%)	
≥12	25	(80.6%)	21	(51.2%)	46	(63.9%)	
CA-125 level at first recurrence, IU/mL, median (range)	68.1	(27.5–110.0)	181.5	(36.5–547.0)	86.6	(30.2–267.8)	0.005 **
Secondary cytoreduction surgery or/and radiotherapy							<0.001 **
No	10	(32.3%)	34	(82.9%)	44	(61.1%)	
Yes	21	(67.7%)	7	(17.1%)	28	(38.9%)	
CA-125 level after 6th cycle of chemotherapy, IU/mL, median (range)	11.1	(7.6–17.7)	14.7	(10.0–43.1)	12.4	(9.5–25.7)	0.093
Maintenance therapy with bevacizumab							0.517
No	19	(61.3%)	22	(53.7%)	41	(56.9%)	
Yes	12	(38.7%)	19	(46.3%)	31	(43.1%)	
Chemotherapy regimens for 1st recurrence							0.227
Platinum plus paclitaxel or PLD	30	(96.8%)	36	(87.8%)	66	(91.7%)	
Others	1	(3.2%)	5	(12.2%)	6	(8.3%)	
Avastin use after recurrence							0.291
No	19	(61.3%)	20	(48.8%)	39	(54.2%)	
Yes	12	(38.7%)	21	(51.2%)	33	(45.8%)	
Complete response after 6th cycle of chemotherapy							<0.001 **
No	4	(12.9%)	21	(51.2%)	25	(34.7%)	
Yes	27	(87.1%)	20	(48.8%)	47	(65.3%)	

Chi-square test or Mann–Whitney U test, median (IQR). * *p* < 0.05, ** *p* < 0.01. ^1^ Debulking surgery.

**Table 2:** Multivariate analyses for PFS in all patients.

PFS						
	Univariate			Multivariable		
	HR	95%CI	*p* Value	HR	95%CI	*p* Value
Age at initial diagnosis, years						
<65	Reference					
≥65	0.61	(0.27–1.36)	0.226			
Histologic type						
Non-serous	Reference					
Serous	1.06	(0.53–2.13)	0.862			
FIGO stage						
I–II	Reference					
III–IV	1.61	(0.78–3.36)	0.201			
Primary treatment						
PDS	Reference					
NAC	1.64	(0.89–3.02)	0.114			
Results of initial DBK						
Optimal	Reference					
Suboptimal	1.05	(0.45–2.46)	0.916			
Platinum-free interval, months						
6–12	Reference			Reference		
≥12	0.09	(0.04–0.20)	<0.001 **	0.12	(0.05–0.29)	<0.001 **
CA-125 level at first recurrence, IU/mL						
<70	Reference					
≥70	1.42	(0.81–2.49)	0.216			
CA-125 level after 6th cycle of chemotherapy, IU/mL						
<35	Reference			Reference		
≥35	3.36	(1.68–6.73)	0.001 **	1.26	(0.49–3.27)	0.633
Secondary cytoreduction surgery or/and radiotherapy						
No	Reference			Reference		
Yes	0.53	(0.30–0.94)	0.029 *	0.81	(0.38–1.72)	0.589
Maintenance therapy with bevacizumab						
No	Reference					
Yes	1.43	(0.82–2.50)	0.208			
Complete response after 6th cycle of chemotherapy						
No	Reference			Reference		
Yes	0.34	(0.18–0.62)	0.001 **	0.64	(0.24–1.67)	0.359
Group						
2nd C/T = 6	Reference					
2nd C/T > 6	1.47	(0.84–2.57)	0.173	0.73	(0.34–1.58)	0.425

Cox proportional hazard regression. * *p* < 0.05, ** *p* < 0.01.

**Table 3:** Multivariate analyses for OS in all patients.

OS						
	Univariate			Multivariable		
	HR	95%CI	*p* Value	HR	95%CI	*p* Value
Age at initial diagnosis, years						
<65	Reference					
≥65	0.48	(0.11–2.04)	0.318			
Histologic type						
Non-serous	Reference					
Serous	0.78	(0.30–2.03)	0.609			
FIGO stage						
I–II	Reference					
III–IV	0.58	(0.13–2.50)	0.465			
Primary treatment						
PDS	Reference					
NAC	1.32	(0.50–3.51)	0.573			
Results of initial DBK						
Optimal	Reference					
Suboptimal	0.29	(0.04–2.14)	0.223			
Platinum-free interval, months						
6–12	Reference			Reference		
≥12	0.20	(0.08–0.51)	0.001 **	0.48	(0.16–1.47)	0.201
CA-125 level at first recurrence, IU/mL						
<70	Reference					
≥70	1.59	(0.65–3.88)	0.305			
CA-125 level after 6th cycle of chemotherapy, IU/mL						
<35	Reference			Reference		
≥35	5.28	(2.05–13.58)	0.001 **	1.05	(0.31–3.62)	0.933
Secondary cytoreduction surgery or/and radiotherapy						
No	Reference			Reference		
Yes	0.27	(0.10–0.75)	0.012 *	0.46	(0.12–1.75)	0.252
Maintenance therapy with bevacizumab						
No	Reference					
Yes	2.25	(0.94–5.39)	0.068			
Complete response after 6th cycle of chemotherapy						
No	Reference			Reference		
Yes	0.13	(0.05–0.37)	<0.001 **	0.21	(0.05–0.92)	0.038 *
Group						
2nd C/T = 6	Reference			Reference		
2nd C/T > 6	2.62	(1.04–6.58)	0.041 *	0.79	(0.22–2.81)	0.722

Cox proportional hazard regression. * *p* < 0.05, ** *p* < 0.01.

**Table 4:** The clinicopathologic characteristics of patients without surgery.

Patients without Surgery (*n* = 44)							
	2nd C/T = 6 (*n* = 10)		2nd C/T > 6 (*n* = 34)		All (*n* = 44)		*p* Value
Age at initial diagnosis, years	57.8	(50.5–67.3)	58.9	(54.2–63.6)	58.9	(53.1–64.4)	0.945
Age at first recurrence, years	59.5	(52.1–70.2)	61.7	(55.8–65.7)	61.7	(54.1–66.4)	0.945
Histologic type							1.000
Non-serous	1	(10.0%)	5	(14.7%)	6	(13.6%)	
Serous	9	(90.0%)	29	(85.3%)	38	(86.4%)	
FIGO stage							0.138
I–II	3	(30.0%)	3	(8.8%)	6	(13.6%)	
III	6	(60.0%)	20	(58.8%)	26	(59.1%)	
IV	1	(10.0%)	11	(32.4%)	12	(27.3%)	
Primary treatment							1.000
PDS	8	(80.0%)	25	(73.5%)	33	(75.0%)	
NAC	2	(20.0%)	9	(26.5%)	11	(25.0%)	
Results of initial DBK							1.000
Optimal	10	(100.0%)	31	(91.2%)	41	(93.2%)	
Suboptimal	0	(0.0%)	3	(8.8%)	3	(6.8%)	
Platinum-free interval, months	12.1	(9.5–25.0)	11.3	(7.5–20.8)	11.3	(7.8–21.3)	0.481
Median (range)							1.000
6–12	5	(50.0%)	18	(52.9%)	23	(52.3%)	
≥12	5	(50.0%)	16	(47.1%)	21	(47.7%)	
CA-125 level at first recurrence, IU/mL, median (range)	75.7	(50.6–119.0)	250.0	(63.9–948.0)	135.0	(59.2–533.3)	0.033 *
Secondary cytoreduction surgery or/and radiotherapy							--
No	10	(100.0%)	34	(100.0%)	44	(100.0%)	
Yes	0	(0.0%)	0	(0.0%)	0	(0.0%)	
CA-125 level after 6th cycle of chemotherapy, IU/mL, median (range)	13.8	(10.7–37.5)	15.7	(10.1–47.8)	15.0	(10.1–46.5)	0.819
Maintenance therapy with bevacizumab							0.723
No	5	(50.0%)	20	(58.8%)	25	(56.8%)	
Yes	5	(50.0%)	14	(41.2%)	19	(43.2%)	
Chemotherapy regimens for 1st recurrence							0.573
Platinum plus paclitaxel or PLD	10	(100.0%)	29	(85.3%)	39	(88.6%)	
Others	0	(0.0%)	5	(14.7%)	5	(11.4%)	
Avastin use after recurrence							1.000
No	5	(50.0%)	19	(55.9%)	24	(54.5%)	
Yes	5	(50.0%)	15	(44.1%)	20	(45.5%)	
Complete response after 6th cycle of chemotherapy							0.150
No	3	(30.0%)	19	(55.9%)	22	(50.0%)	
Yes	7	(70.0%)	15	(44.1%)	22	(50.0%)	

Chi-square test or Mann–Whitney U test, median (IQR). * *p* < 0.05.

**Table 5:** Multivariate analyses for PFS in patients without surgery.

PFS (Patients without Surgery)						
	Univariate			Multivariable		
	HR	95%CI	*p* Value	HR	95%CI	*p* Value
Age at initial diagnosis, years						
<65	Reference					
≥65	0.75	(0.32–1.74)	0.500			
Histologic type						
Non-serous	Reference					
Serous	0.85	(0.33–2.23)	0.748			
FIGO stage						
I–II	Reference					
III–IV	1.75	(0.65–4.76)	0.270			
Primary treatment						
PDS	Reference					
NAC	1.83	(0.88–3.81)	0.106			
Results of initial DBK						
Optimal	Reference					
Suboptimal	0.79	(0.19–3.33)	0.747			
Platinum-free interval, months						
6–12	Reference			Reference		
≥12	0.14	(0.05–0.37)	<0.001 **	0.18	(0.06–0.48)	0.001 **
CA-125 level at first recurrence, IU/mL						
<70	Reference			Reference		
≥70	1.95	(0.87–4.40)	0.107	1.47	(0.62–3.51)	0.384
CA-125 level after 6th cycle of chemotherapy, IU/mL						
<35	Reference			Reference		
≥35	2.46	(1.13–5.37)	0.024 *	0.81	(0.27–2.50)	0.728
Secondary cytoreduction surgery or/and radiotherapy						
No	Reference					
Yes	--					
Maintenance therapy with bevacizumab						
No	Reference					
Yes	1.35	(0.67–2.72)	0.403			
Complete response after 6th cycle of chemotherapy						
No	Reference			Reference		
Yes	0.38	(0.18–0.80)	0.011 *	0.76	(0.27–2.09)	0.590
Group						
2nd C/T = 6	Reference					
2nd C/T > 6	0.95	(0.43–2.07)	0.893			

Cox proportional hazard regression. * *p* < 0.05, ** *p* < 0.01.

**Table 6:** Multivariate analyses for OS in patients without surgery.

OS (Patients without Surgery)						
	Univariate			Multivariable		
	HR	95%CI	*p* Value	HR	95%CI	*p* Value
Age at initial diagnosis, years						
<65	Reference					
≥65	0.48	(0.11–2.15)	0.341			
Histologic type						
Non-serous	Reference					
Serous	0.39	(0.12–1.26)	0.116			
FIGO stage						
I–II	Reference					
III–IV	1.17	(0.31–4.38)	0.821			
Primary treatment						
PDS	Reference					
NAC	1.49	(0.52–4.24)	0.458			
Results of initial DBK						
Optimal	Reference					
Suboptimal	0.04	(0.00–177.14)	0.460			
Platinum-free interval, months						
6–12	Reference			Reference		
≥12	0.27	(0.09–0.81)	0.019 *	0.50	(0.15–1.63)	0.249
CA-125 level at first recurrence, IU/mL						
<70	Reference			Reference		
≥70	3.22	(0.90–11.54)	0.073	1.80	(0.45–7.23)	0.408
CA-125 level after 6th cycle of chemotherapy, IU/mL						
<35	Reference			Reference		
≥35	4.09	(1.46–11.48)	0.007 **	1.17	(0.29–4.71)	0.829
Secondary cytoreduction surgery or/and radiotherapy						
No	Reference					
Yes	--					
Maintenance therapy with bevacizumab						
No	Reference					
Yes	2.27	(0.82–6.31)	0.116			
Complete response after 6th cycle of chemotherapy						
No	Reference			Reference		
Yes	0.18	(0.06–0.58)	0.004 **	0.32	(0.07–1.55)	0.157
Group						
2nd C/T = 6	Reference					
2nd C/T > 6	1.16	(0.37–3.62)	0.799			

Cox proportional hazard regression. * *p* < 0.05, ** *p* < 0.01.

**Table 7:** Adverse effects in all patients.

	Grade 1–2					Grade 3–4				
	CT = 6 (*n* = 31)		CT > 6 (*n* = 41)		*p* Value	CT = 6 (*n* = 31)		CT > 6 (*n* = 41)		*p* Value
Anemia	16	(51.6%)	31	(75.6%)	0.034 *	10	(32.3%)	10	(24.4%)	0.460
Neutropenia	13	(52.0%)	17	(43.6%)	0.511	12	(48.0%)	22	(56.4%)	0.511
Thrombocytopenia	8	(47.1%)	13	(43.3%)	0.805	9	(52.9%)	17	(56.7%)	0.805
Liver toxicity	5	(83.3%)	3	(75.0%)	1.000	1	(16.7%)	1	(25.0%)	1.000
Renal toxicity	12	(92.3%)	17	(85.0%)	1.000	1	(7.7%)	3	(15.0%)	1.000

Chi-square test. * *p* < 0.05.

## Data Availability

Data are available upon reasonable request. The datasets used during the current study are available from the Taichung Veterans General Hospital; however, restrictions apply regarding the availability of these data, as they are not publicly available. Nevertheless, the data are available from the corresponding author upon reasonable request and with permission from the Taichung Veterans General Hospital.
